# An Improved Method for Quick Quantification of Unsaturated Transferrin

**DOI:** 10.3390/bios12090708

**Published:** 2022-09-01

**Authors:** Ruirui Guo, Juanjuan Gao, Lingyun Hui, Yanqing Li, Junhui Liu, Yao Fu, Lei Shi, Yawen Wang, Bing Liu

**Affiliations:** 1 BioBank, The First Affiliated Hospital of Xi’an Jiaotong University, Xi’an 710061, China; 2 Department of Laboratory Medicine, The First Affiliated Hospital of Xi’an Jiaotong University, Xi’an 710061, China; 3 College of Chemical Engineering, North China University of Science and Technology, Tangshan 063210, China

**Keywords:** blood test, MTC, UIBC, transferrin, transferrin saturation, hepatic cancer, visual indicator

## Abstract

Blood iron levels play a vital role in oxygen metabolism and energy generation whilst transporter protein, transferrin, binds and delivers iron to the transferrin receptor of endosomal compartments of cells. Consequently, the iron-binding capacity of transferrin is an important indicator for many diseases, and its measurements are used in the diagnosis and treatment of anaemias. Various assays, including Total Iron Binding Capacity (TIBC), Unsaturated Iron-Binding Capacity (UIBC) and Transferrin Saturation (TS), were developed to assess the iron-binding capacity of transferrin. Clinically, UIBC is measured in serum by a multi-step liquid ferrozine method and subjected to interference from conditions such as haemolysis and lipemia. Here, we report a quick method that directly measures the concentration of apotransferrin in EDTA-treated plasma, theoretically equivalent to UIBC. Importantly, this supramolecular assembly-based method is more time-efficient, cost-effective and insensitive to serum cation fluctuations. With additional colorimetric property, this method also provides a visual indicator for abnormal health conditions with extreme transferrin statuses such as those found in cancers. Its minimal requirement for equipment would be particularly useful for diagnosis in remote and under-developed regions.

## 1. Introduction

Iron is an essential element for almost all organisms, including humans, in which the level is tightly controlled throughout almost all biological processes [1]. It is delivered to tissues by circulating transferrin and captured by transferrin receptors of the destination cells [2]. The role of transferrin is highlighted in the congenital atransferrinemia/hypotransferrinemia characterized by hypochromic, microcytic anemia and hemosiderosis, in which the body fails to produce enough functional transferrin, i.e., mutation in transferrin (*TF*) gene [3,4].

Iron status can be assessed by several natural serum-based indicators, including serum ferritin, transferrin, and transferrin receptor [5]. While an abnormal serum ferritin level indicates iron storage disorder, both transferrin and soluble transferrin receptor (sTfR) tests are used to diagnose iron-deficiency anaemia or overload [6,7]. Status of serum transferrin can be assessed by a few available laboratory tests, including serum iron, total iron-binding capacity (TIBC), transferrin saturation (TS) and unsaturated iron-binding capacity (UIBC) [8]. While TIBC measures the blood’s capacity to bind iron with transferrin, TS measures the percentage of iron occupied transferrin, which is calculated using the serum iron divided by TIBC. UIBC is an inexpensive, multi-step method alternative to transferrin saturation, which measures the vacant iron-binding capacity in transferrin. Ferrozine is a popular regent used in these tests as it is capable of forming complex with iron, which is detectable and quantifiable colorimetrically [9]. Among the three transferrin tests, the process of ferrozine-based UIBC is easily automated in most clinical analyzers and is also the preferred method over TIBC and serum iron tests in practice [10]. However, conditions such as haemolysis which alter the serum content and liver diseases such as cirrhosis and hepatitis with altered transferrin production may have strong influences on UIBC results [11,12]. Furthermore, UIBC was found to be less reliable than other tests due to high standard deviations (SDs) [13]. Overall, the diagnosis of iron-deficiency anaemia is still very challenging, as the above serum-based methods can be complicated and interfered with by many factors [10,14].

Here, we report a method based on the supramolecular complex between 3,3′-disulfopropyl-4,5,4′,5′-dibenzo-9-methyl-thiacarbocyanine triethylammonium salt (MTC) and apotransferrin (apo-Tf), whose formation directly reflects the concentration of apotransferrin [15,16]. Due to the nature of this interaction and the use of EDTA as a sequester, abnormal serum conditions such as haemolysis have no profound effect on this method [17]. Based on our comparative study on over 500 blood samples, this quick method is more consistent than UIBC in healthy participants. Additionally, this test is accompanied by visible colour changes, a feature with the potential to be used in remote clinics with simple setups.

## 2. Materials and Methods

### 2.1. Sample Collection and Preparation

A total of 523 plasma (EDTA as the anticoagulant) and paired serum samples from participants were collected from inpatients and health examination population in the First Affiliated Hospital of Xi’an Jiaotong University from September 2020 to December 2021. Among them, 253 were used for preliminary experiments and the rest for the large-scale comparison. Ethical approval for this study was obtained from the Institutional Review Board of the First Affiliated Hospital of Xi’an Jiaotong University with the approval number: XJTU1AF2020LSK-279.

### 2.2. MTC Test

A 2 mM MTC solution was prepared by adding 0.0076 g MTC to 5 mL deionized water. The relevant amount of apo-Tf (Sigma, Sigma Aldrich (Shanghai) Trading Co., Ltd., Shanghai, China) was added to 20 mM Tris-HCl, pH 7 solution for theoretical analysis, and plasma samples were diluted with water to appropriate concentration. Next, 100 μL MTC solution and 500 μL samples were mixed and incubated for 5 min before measuring the absorbance by CYTATION 5 imaging reader (BioTek, American Berten Instrument Co., Ltd. Beijing Representative Office, Beijing, China) from 350 to 700 nm in three duplicated wells.

### 2.3. UIBC Test

UIBC tests were performed manually by either following Tietz textbook of clinical chemistry [18] or using UIBC liquid kit (Sentinel Diagnostics, Milano, Italy) on Architect c16000 analyzer (Abbott diagnosis, Abbott Trading (Shanghai) Co., Ltd., Shanghai, China).

### 2.4. Result Analysis

Mann–Whitney U test and Fisher’s exact test were used for comparisons between two groups, and Games–Howell test was for multiple comparisons. Statistical analysis was conducted using SPSS 21.0 (SPSS, Chicago, IL, USA) and two-sided *p* values < 0.05 were considered statistically significant.

## 3. Results

### 3.1. Inconsistency in the UIBC Measurement

**Figure 1:**
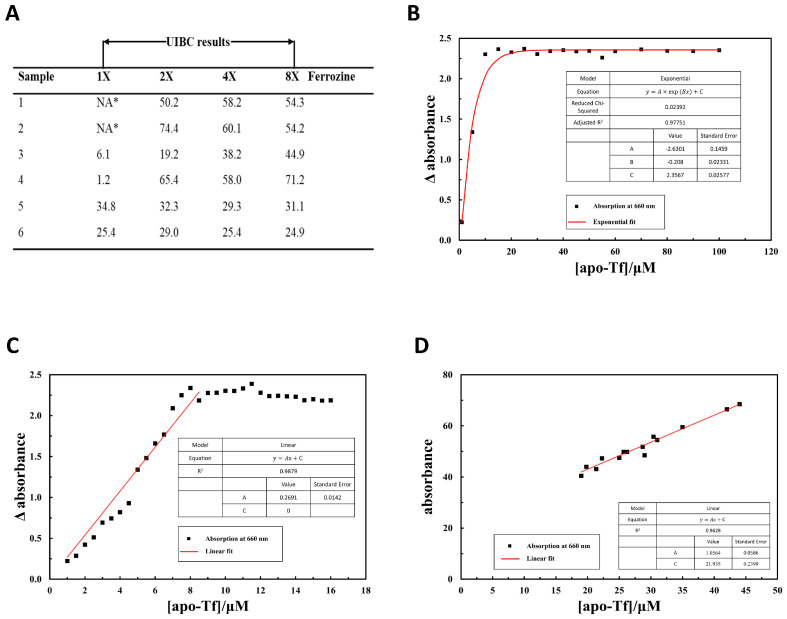
MTC assay. (**A**) Inconsistency in UIBC at low readings. A 0.04 mL volume of 7.8 mM ferrozine was used in the standard UIBC test (1×). Additionally, up to 8-fold levels of ferrozine were used in the comparative study. UIBC results were derived by UIBC (µmol/L) = 89 (µmol/L Iron added) − Excess Iron (µmol/L). NA* indicates for a negative result. (**B**) The graph of absorbance at 660 nm against the concentration of apo-Tf in Tris-HCL solution, using MTC as the probe. The graph follows an exponential fit shown as y=A×exp(Bx)+C, where *y* is the absorbance at 660 nm, *x* is the concentration of apo-Tf, *A* = −2.63, *B* = −0.21 and *C* = 2.36. The graph shows a potential linear correlation for apo-Tf under 10 μM concentration. (**C**) The graph of absorbance at 660 nm against the concentration of apo-Tf in Tris-HCL solution, using MTC as the probe, for apo-Tf concentration ranging from 0 to 16 μM. The graph shows a linear correlation between 1 to 8 μM shown as y=Ax+C, where *y* is the absorbance at 660 nm, *x* is the concentration of apo-Tf, *A* = 0.27, *C* = 0. (**D**) The graph of absorbance at 660 nm against the concentration of apo-Tf in plasma sample diluted 50 times, using MTC as the probe. The data on the graph follow a linear correlation shown as y=Ax+C where *y* is the absorbance at 660 nm, *x* is the concentration of apo-Tf, *A* = 0.022, *C* = 0.44.

In the routine UIBC measurements using a 96-well plate in a standard spectrophotometer, we frequently came across zero readings, which indicate that serum abnormalities may hinder the test. To verify these results, we selected a few samples with both out-of-range and normal readings, and performed the UIBC assay manually (Figure 1A). The manual results agreed with those generated from the automatic plate reader, with zero readings for the first two, low numbers for the middle ones and moderate readings for the last two (1× ferrozine sample in the first column). However, the results for the first four samples changed dramatically while the last two remained largely undisturbed, when more ferrozine was used in the assay. As ferrozine does not interfere with the absorbance measurement and UIBC reading is independent from amount of ferrozine used, the results suggest certain serum conditions may greatly affect UIBC results. More importantly, the first four samples which have inconsistent results when using different amounts of ferrozine, were all sourced from hepatic cancer patients. As UIBC results were previously reported as abnormally small when transferrin was highly saturated with iron [13], our results also suggest that UIBC is inaccurate for zero or low readings and hepatic cancer may have a role in this inconsistency.

### 3.2. Establishing a Linear Correlation between A660 and Concentration of Apotransferrin in Non-Biological Solutions

To find a more accurate alternative way for testing transferrin, we chose Cyanine dye MTC as the candidate due to its interaction with transferrin [16]. MTC distinguishes apo- (iron-free) and holo-(iron-bound) transferrin due to significant differences in their three-dimensional structures, as a large hydrophobic patches exposed on a semi-opened jaw of the apo-Tf are absent from the holoprotein [15,16], thus being a potential indicator for the saturation of transferrin. To identify whether MTC is feasible for clinical applications, we first tested its suitability as an indicator in non-biological conditions before propagating to blood samples. We performed the experiment in Tris-HCl buffer and plotted the absorption at 660 nm (A660) against the concentration of apo-Tf (Figure 1B). The curve matches an exponential fit with a potential linear correlation under 10 μM region. To verify this possible linear correlation, we repeated the experiment with more increments in 0–16 μM range and established a linear correlation between the A660 and concentration of apo-Tf at 0–8 μM (Figure 1C). This simple correlation allows the quick quantification of concentration of apo-Tf using A660 in the non-biological solution.

### 3.3. The Extension of MTC Method to Blood Plasma

As interferences may arise from serum biomolecules and cations, the MTC protocol derived from non-biological solution may not be directly applied to the more complicated blood samples. As MTC may also interact with other proteins, transferrin must have priority to interact with MTC over other serum proteins. We then compared the major protein components in the serum and found Apo-Tf had the most extreme surface exposed hydrophobic surface (data not shown) which is more likely to have priority to interact with MTC [16]. To eliminate any interference from serum cations, we utilized the EDTA-treated plasma, a common intermediate in the routine blood test, for the MTC experiment. EDTA is commonly used as an anticoagulant in medical practices, which also conveniently benefits this method as MTC is very sensitive to cations [19].

To test whether MTC could be used in EDTA-treated blood samples, we selected 14 blood samples from healthy individuals whose blood tests showed no abnormality. We then compared the results between ferrozine-based UIBC using serum samples and A660 readings in MTC test, which were obtained from four parallel plasma samples at 5, 20, 50- and 100-times dilutions. Based on our preliminary results, we chose 2 mM MTC and 50-times diluted plasma for further analysis (Figure S1, Supplementary Materials). First, the theoretical concentration of serum apo-Tf at 50-times dilution falls in the linear correlation range as described in non-biological solution. More importantly, we managed to establish a linear correlation between concentration of apo-Tf obtained from UIBC test and A660 reading from MTC test (Figure 1D). As the blank sample (plasma without apo-Tf) is not measured, we estimated it to be 0.44 from the linear correlation: Conc. apo-Tf = (A660 − 0.44) ∗ 50.

### 3.4. Systemic Comparison

**Figure 2:**
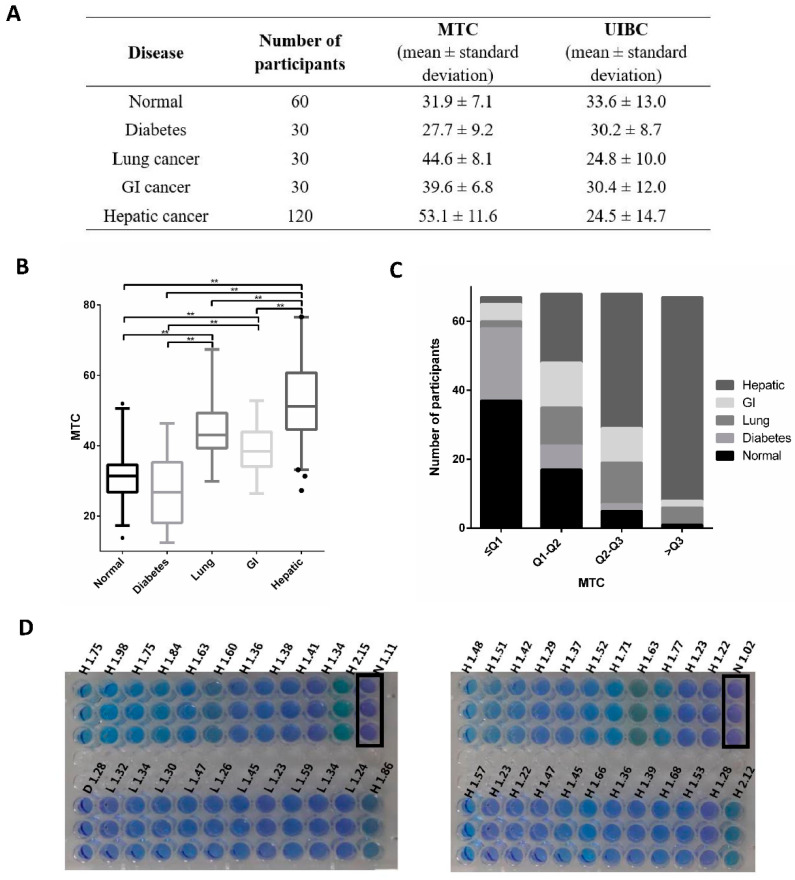
Systemic comparison between MTC and UIBC. (**A**) Comparison of concentration of apo-Tf obtained from MTC and UIBC for samples with different diseases and their corresponding sample size. Hepatic-disease patients were found with the highest concentration of apo-Tf obtained from MTC assay. (**B**) Distribution of MTC among normal subjects and patients with different types of disease. The boxes indicate interquartile ranges (IQRs) and the median (middle line). Whiskers extend to the most extreme points within 1.5-fold IQR. Outliers are plotted individually as dots. ** represents *p* values derived from Games–Howell test for multiple comparisons that are less than 0.001. (**C**) Distribution of subjects with different diseases in four quartile intervals of apo-Tf obtained from MTC assay. Q1, Q2 and Q3 illustrate the first (33.296), second (41.767) and third quartile (51.783) of MTC, respectively. (**D**) Colorimetric feature of MTC assay. Samples from normal group (abbreviated as N and highlighted by black square) shows purple color due to the lower A660 reading. In contrast, samples from patients with hepatic cancer (abbreviated as H) have higher A660 reading which resulted in more green colors. Lung cancer and diabetic samples are labelled as L and D, respectively.

**Figure 3:**
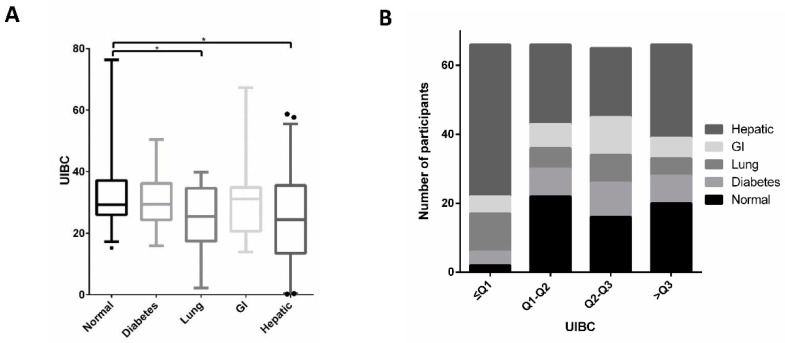
UIBC in different disease groups. (**A**) Distribution of UIBC among normal subjects and patients with different types of disease. The boxes indicate interquartile ranges (IQRs) and the median (middle line). Whiskers extend to the most extreme points within 1.5-fold IQR. Outliers are plotted individually as dots. * represents *p* values derived from Games–Howell test for multiple comparisons that are less than 0.05. (**B**) Distribution of subjects with different diseases in four quartile intervals of apo-Tf obtained from UIBC assay. Q1, Q2 and Q3 illustrate the first (19.369), second (27.287) and third quartiles (36.057) of UIBC, respectively.

To evaluate the MTC method and conduct a systemic comparison with UIBC, we collected 270 samples, of which 60 were plasma samples from healthy individuals and 210 from patients with diabetes, and lung, gastrointestinal (GI) or hepatic cancers (Figure 2A). In general, the readings from the MTC method were in line with UIBC in normal and diabetes groups (Figure 2, Figure 3 and Figure S4). The discrepancy between the two methods resides in the cancer groups, in which hepatic cancer group is significantly different compared with normal group. With 120 participants, the hepatic group is statistically distinctive from others (Figure 2B, Figure 3A and Figures S2a and S3a). Moreover, MTC result for lung and gastrointestinal cancer patients was significantly larger than normal cases (*p* < 0.001) while no statistical difference between them (*p* = 0.084, Figure S2a). Furthermore, we categorized MTC and UIBC into four groups and determined the distribution of different samples in each group, respectively, in which hepatic-disease samples tested by MTC occupy most of the fourth quartile (88.1%) (Figure 2C and Figure S2b), while the samples tested by UIBC account for 66.7% of the first quartile (Figure 3B and Figure S3b). Additionally, while MTC in males and females showed no significant differences (Table 1), female diabetics had larger UIBC results (*p* = 0.033, Table 2).

Notably, there is a significant number of low or negative readings in the hepatic cancer group, which theoretically corresponds to ultra-low unsaturated iron-binding capacity in TF. We suspected that complex blood conditions in these patients may interfere with ferrozine used in UIBC as the MTC method clearly distinguishes them as hepatic cancer patients. Interestingly, the UIBC readings for these hepatic cancer patients increased dramatically to the level of MTC when more was ferrozine used, while those for the normal patients remain relatively unchanged (Figure 1A). Theoretically, UIBC reading is independent from the amount of ferrozine used as observed in the samples from our normal group. This inconsistency in UIBC test is likely due to the interference from other cations such as ferric ions [20].

### 3.5. Visible Indicator

As the MTC reaction mixture displays colour variations at different A660, the hepatic cancer group, which generally had higher readings, showed a more brownish colour compared with the purple in the normal group (Figure 2D and Figure S5). This property allows quick visual diagnosis of abnormalities including hepatic cancer using a minimal set of equipment, which would potentially benefit remote clinics with limited access to resources.

## 4. Discussion and Conclusions

Our method utilizes the MTC as a probe to assess the concentration of apo-Tf while UIBC uses external iron to occupy the binding capacity of apo-Tf and ferrozine to sequester the remaining iron. As ferrozine is subject to interferences from other serum metal cations, UIBC is reported to be less reliable compared with other blood tests [13]. Cyanine dyes such as MTC have been used to probe DNA, RNA and protein structures [21,22], and its application in tests that assess the states of transferrin addresses two major sources of interferences in plasma–metal cations and other proteins. Firstly, MTC method uses EDTA-treated plasma, in which anticoagulant EDTA sequesters metals and minimizes the interference from cations. In contrast, the ferrozine-based UIBC methods use ferrozine to calculate the excess amount of iron added in the blood sample, during which anticoagulant should not be used. Secondly, MTC is likely to interact with apo-Tf first due to its extreme surface geometry and hydrophobic patch compared with other major serum proteins [16]. Thus, MTC prioritizes its interaction with apo-Tf at low concentration, theoretically.

MTC method is not identical to UIBC as apo-Tf does not necessarily show theoretical iron-binding capacity due to complicated serum-iron conditions. In our comparative study, UIBC is lower in cancer patients, mainly because of the abnormal low or negative readings (Figure 1A and Figure S4). Although such readings may be an indicator of iron overload, the UBIC reading turned out much larger numbers close to the reading of MTC when more ferrozine was used. Theoretically, the final results should be the same regardless of the amount of ferrozine added as seen in the normal group. As ferrozine interacts with more than just ferrous ions [20], this inconsistency in UIBC is likely due to the complexity during cancer development.

**Figure 4:**
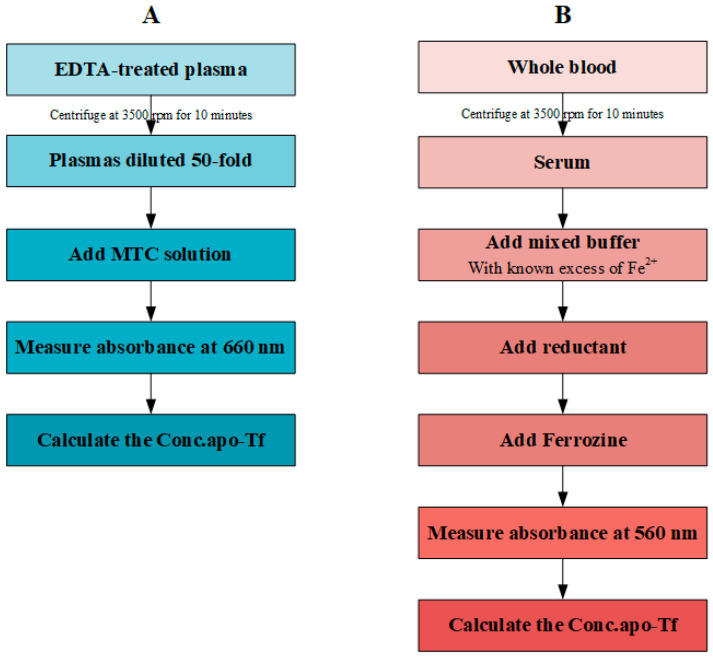
Standard procedures for MTC and UIBC. (**A**) 5-step protocol for MTC test. (**B**) 7-step protocol for MTC test.

While providing the more consistent reading, our MTC method further simplifies the protocol (Figure 4). With one dilution step and one single reagent, our method is more time-efficient and easier to perform either automatically or manually. Furthermore, the obvious colour change in hepatic cancer patients allows visual diagnosis of the disease when spectrometers are not available. Therefore, the MTC method directly quantifies the complex formation between apo-Tf in EDTA-treated plasma, providing advantages in complex blood conditions. The simplified protocol is more cost- and time-effective compared to the current ferrozine-based method. Additionally, its colorimetric feature allows for quick visual indication of hepatic cancer.

## 5. Patents

The authors have filled the following patent application related to this work: application number 202010845618.3 on clinical application of MTC in blood test (inventors: B.L., Y.W., Y.D., and R.G.).

## Figures and Tables

**Table 1:** Comparisons of MTC between males and females in each disease group.

Disease Group	Male		Female		*p* ^1^
	Number of Participants	MTC (Mean ± Standard Deviation)	Number of Participants	MTC (Mean ± Standard Deviation)	
Normal	33	31.2 ± 6.0	27	32.9 ± 8.2	0.453
Diabetes	23	26.6 ± 8.5	7	31.2 ± 11.1	0.413
Lung cancer	23	45.7 ± 7.4	7	41.1 ± 9.9	0.288
GI cancer	21	39.1 ± 6.8	9	40.8 ± 7.1	0.397
Hepatic cancer	89	52.9 ± 10.8	31	53.6 ± 13.8	0.995

^1^ Comparisons were performed by Mann–Whitney U test.

**Table 2:** Comparisons of UIBC between males and females in each disease group.

Disease Group	Male		Female		*p* ^1^
	Number of Participants	UIBC (Mean ± Standard Deviation)	Number of Participants	UIBC (Mean ± Standard Deviation)	
Normal	33	30.2 ± 8.3	27	37.7 ± 16.4	0.115
Diabetes	23	28.2 ± 7.3	7	36.9 ± 9.9	0.033
Lung cancer	23	23.3 ± 10.1	7	29.7 ± 8.7	0.144
GI cancer	20	29.7 ± 10.0	9	32.0 ± 16.1	0.982
Hepatic cancer	84	23.1 ± 14.3	30	28.5 ± 15.4	0.059

^1^ Comparisons were performed by Mann–Whitney U test.

## Data Availability

Not applicable.

## Supplementary Materials

The following supporting information can be downloaded at: https://www.mdpi.com/article/10.3390/bios12090708/s1, Figure S1: Determination of the concentration of MTC used in the test; Figure S2: Distribution of MTC in different disease groups; Figure S3: Distribution of UIBC in different disease groups; Figure S4: Systemic comparison between MTC and UIBC; Figure S5: MTC reaction colors.
