# Supplementary material for: An Improved Method for Quick Quantification of Unsaturated Transferrin
Source: Biosensors (Basel). 2022 Sep 1;12(9):708. doi: 10.3390/bios12090708 (PMC9496074; doi:10.3390/bios12090708)
Supplement: Supplementary file 1 [file biosensors-12-00708-s001.zip › biosensors-1856168-supplementary.pdf]

Supporting Information

Title: An Improved Method for Quick Quantification of Unsaturated Transferrin

Authors: Ruirui Guo, Juanjuan Gao, Lingyun Hui, Yanqing Li, Junhui Liu, Yao Fu, Lei Shi, Yawen Wang and Bing Liu

Contents:

Supporting Information 1
Figure S1. Determination of the concentration of MTC used in the test 2
Figure S2. Distribution of MTC in different disease groups 3
Figure S3. Distribution of UIBC in different disease groups 4
Figure S4. Systemic comparison between MTC and UIBC 5
Figure S5. MTC reaction colors 11

Figure S1. Determination of the concentration of MTC used in the test.

2 µl of MTC at 0.5 mM, 1mM or 2 mM was added to 98 µl of plasma at 10X, 20X 50X or 100X dilution in each reaction. The criteria for choosing the concentration are to minimize the usage of MTC while keeping the color appealing to naked eyes. The arrow indicates the 2 mM of MTC and 50 times diluted plasma was chosen for the reaction.

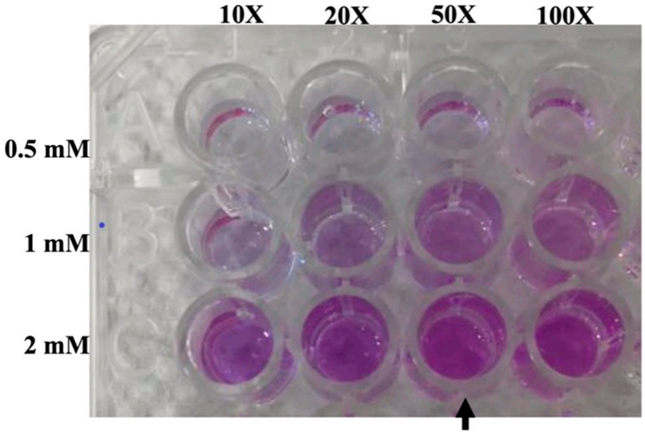

Figure S2. Distribution of MTC in different disease groups.

a: Comparisons of MTC in different disease groups.

**Reference group**	**Comparison group**	***P*** [a]
Normal	Diabetes	0.186
Normal	Lung cancer	**<0.001**
Normal	GI cancer	**<0.001**
Normal	Hepatic cancer	**<0.001**
Hepatic cancer	Diabetes	**<0.001**
Hepatic cancer	Lung cancer	**<0.001**
Hepatic cancer	GI cancer	**<0.001**
Lung cancer	Diabetes	**<0.001**
Lung cancer	GI cancer	0.084
GI cancer	Diabetes	**<0.001**

[a] Multiple comparison between two groups were performed by Games-Howell test.

b: Distribution of different diseases among four quartile intervals of MTC.

**Disease group**	**MTC**, n (%)				*P* [b]
	≤Q1 [a]	Q1-Q2 [a]	Q2-Q3 [a]	>Q3 [a]	
Normal	37 (61.7)	17 (28.3)	5 (8.3)	1 (1.7)	-
Diabetes	21 (70.0)	7 (23.3)	2 (6.7)	0 (0.0)	0.883
Lung cancer	2 (6.7)	11 (36.7)	12 (40.0)	5 (16.7)	**<0.001**
GI cancer	5 (16.7)	13 (43.3)	10 (33.3)	2 (6.7)	**<0.001**
Hepatic cancer	2 (1.7)	20 (16.7)	39 (32.5)	59 (49.2)	**<0.001**

[a] Q1, Q2 and Q3 represent the first (33.296), second (41.767) and third quartile (51.783) of MTC, respectively.

[b] Fisher's exact test was used for comparisons between each disease group and the normal group.

Figure S3. Distribution of UIBC in different disease groups.

a: Comparisons of UIBC in different disease groups.

Reference group	Comparison group	*P* [a]
Normal	Diabetes	0.595
Normal	Lung cancer	**0.006**
Normal	GI cancer	0.779
Normal	Hepatic disease	**0.001**
Hepatic cancer	Diabetes	0.061
Hepatic cancer	Lung cancer	>0.999
Hepatic cancer	GI cancer	0.183
Lung cancer	Diabetes	0.178
Lung cancer	GI cancer	0.309
GI cancer	Diabetes	>0.999

[a] Multiple comparison between two groups were performed by Games-Howell test.

b: Distribution of different diseases among four quartile intervals of UIBC.

Disease group	**UIBC**, n (%)				*P* [b]
	≤Q1 [a]	Q1-Q2 [a]	Q2-Q3 [a]	>Q3 [a]	
Normal	2 (3.3)	22 (36.7)	16 (26.7)	20 (33.3)	-
Diabetes	4 (13.3)	8 (26.7)	10 (33.3)	8 (26.7)	0.260
Lung cancer	11 (36.7)	6 (20.0)	8 (26.7)	5 (16.7)	**<0.001**
GI cancer	5 (17.2)	7 (24.1)	11 (37.9)	6 (20.7)	0.060
Hepatic cancer	44 (38.6)	23 (20.2)	20 (17.5)	27 (23.7)	**<0.001**

[a] Q1, Q2 and Q3 represent the first (19.369), second (27.287) and third quartile (36.057) of UIBC, respectively.

[b] Fisher's exact test was used for comparisons between each disease group and the normal group.

Figure S4. Systemic comparison between MTC and UIBC.

ID	Group	OD_{660}	MTC	UIBC
1	Normal	1.021	29.067	23.014
2	Normal	1.007	28.350	31.635
3	Normal	0.997	27.833	25.712
4	Normal	1.101	33.067	38.381
5	Normal	0.929	24.450	29.086
6	Normal	1.106	33.283	36.057
7	Normal	1.144	35.183	22.789
8	Normal	0.868	21.383	26.312
9	Normal	0.943	25.133	49.326
10	Normal	0.891	22.550	29.910
11	Normal	0.880	22.000	19.790
12	Normal	1.097	32.833	21.140
13	Normal	1.208	38.417	25.787
14	Normal	0.861	21.050	21.365
15	Normal	1.060	31.017	15.218
16	Normal	0.965	26.250	34.408
17	Normal	1.293	42.633	33.734
18	Normal	0.885	22.233	32.159
19	Normal	1.133	34.633	51.425
20	Normal	1.127	34.333	23.239
21	Normal	1.021	29.067	37.182
22	Normal	1.119	33.950	30.435
23	Normal	0.947	25.350	22.264
24	Normal	1.068	31.400	36.282
25	Normal	1.112	33.600	19.041
26	Normal	1.327	44.367	29.461
27	Normal	1.077	31.833	26.462
28	Normal	1.140	35.017	28.711
29	Normal	1.055	30.733	66.343
30	Normal	0.997	27.833	26.237
31	Normal	1.059	30.933	36.282
32	Normal	0.959	25.967	25.937
33	Normal	0.929	24.450	36.282
34	Normal	1.033	29.650	39.431
35	Normal	0.936	24.800	28.486
36	Normal	1.036	29.800	28.711
37	Normal	1.112	33.600	66.642
38	Normal	0.983	27.133	37.707
39	Normal	1.196	37.800	29.910
40	Normal	1.106	33.300	58.546
41	Normal	1.290	42.500	26.987
42	Normal	1.005	28.233	47.677
43	Normal	1.319	43.933	57.647
44	Normal	1.015	28.767	34.783
45	Normal	1.148	35.400	26.612
46	Normal	1.435	49.750	36.882
47	Normal	1.127	34.350	22.414

48	Normal	1.206	38.300	43.629
49	Normal	1.253	40.667	27.287
50	Normal	0.978	26.883	26.387
51	Normal	1.093	32.667	43.104
52	Normal	1.075	31.767	27.137
53	Normal	1.086	32.283	33.958
54	Normal	1.166	36.283	28.336
55	Normal	1.489	52.450	23.763
56	Normal	1.275	41.750	24.813
57	Normal	1.142	35.117	41.080
58	Normal	1.078	31.883	28.111
59	Normal	1.084	32.200	27.062
60	Normal	0.726	14.283	87.108
61	GI cancer	1.505	53.267	41.803
62	GI cancer	1.023	29.167	28.791
63	GI cancer	1.279	41.967	31.558
64	GI cancer	1.183	37.167	13.909
65	GI cancer	1.072	31.583	NA*
66	GI cancer	1.491	52.550	17.349
67	GI cancer	1.132	34.600	21.163
68	GI cancer	1.322	44.083	44.346
69	GI cancer	1.112	33.617	52.347
70	GI cancer	1.296	42.817	27.370
71	GI cancer	1.129	34.467	35.746
72	GI cancer	1.253	40.633	31.259
73	GI cancer	1.161	36.033	31.109
74	GI cancer	1.299	42.967	19.369
75	GI cancer	1.103	33.133	21.911
76	GI cancer	0.979	26.950	33.951
77	GI cancer	1.164	36.183	24.603
78	GI cancer	1.137	34.850	20.116
79	GI cancer	1.377	46.867	37.167
80	GI cancer	1.392	47.583	31.109
81	GI cancer	1.433	49.650	67.304
82	GI cancer	1.346	45.283	16.228
83	GI cancer	1.130	34.517	19.369
84	GI cancer	1.228	39.383	33.951
85	GI cancer	1.308	43.400	31.782
86	GI cancer	1.083	32.150	19.892
87	GI cancer	1.187	37.333	24.528
88	GI cancer	1.223	39.133	23.108
89	GI cancer	1.212	38.617	48.833
90	GI cancer	1.408	48.383	31.334
91	Diabetes	0.819	18.950	24.813
92	Diabetes	0.774	16.717	33.584
93	Diabetes	0.781	17.050	22.939
94	Diabetes	0.926	24.317	24.963
95	Diabetes	0.966	26.300	41.605
96	Diabetes	0.791	17.567	32.759
97	Diabetes	0.727	14.333	27.362
98	Diabetes	0.708	13.417	26.087

99	Diabetes	0.767	16.350	35.383
100	Diabetes	0.698	12.900	32.609
101	Diabetes	0.983	27.150	17.616
102	Diabetes	0.916	23.817	36.432
103	Diabetes	0.941	25.033	15.892
104	Diabetes	0.985	27.267	19.266
105	Diabetes	1.056	30.817	39.731
106	Diabetes	1.180	37.000	27.287
107	Diabetes	0.983	27.133	36.132
108	Diabetes	0.890	22.517	42.654
109	Diabetes	1.053	30.633	25.637
110	Diabetes	1.042	30.117	21.290
111	Diabetes	1.151	35.567	16.267
112	Diabetes	1.168	36.417	38.156
113	Diabetes	1.118	33.883	29.086
114	Diabetes	1.038	29.900	22.414
115	Diabetes	1.183	37.150	50.450
116	Diabetes	1.378	46.883	27.961
117	Diabetes	1.273	41.650	44.004
118	Diabetes	1.276	41.783	29.985
119	Diabetes	1.206	38.283	35.008
120	Diabetes	1.016	28.817	29.760
121	Lung cancer	1.321	44.050	18.695
122	Lung cancer	1.342	45.100	27.520
123	Lung cancer	1.296	42.783	22.061
124	Lung cancer	1.469	51.467	14.657
125	Lung cancer	1.263	41.133	13.760
126	Lung cancer	1.453	50.650	25.575
127	Lung cancer	1.231	39.533	15.704
128	Lung cancer	1.587	57.350	25.351
129	Lung cancer	1.336	44.817	36.120
130	Lung cancer	1.237	39.867	31.109
131	Lung cancer	1.164	36.200	39.709
132	Lung cancer	1.253	40.667	18.247
133	Lung cancer	1.322	44.100	17.723
134	Lung cancer	1.516	53.800	27.146
135	Lung cancer	1.251	40.533	29.389
136	Lung cancer	1.395	47.733	21.238
137	Lung cancer	1.144	35.217	38.064
138	Lung cancer	1.274	41.683	38.214
139	Lung cancer	1.425	49.250	18.396
140	Lung cancer	1.797	67.850	8.600
141	Lung cancer	1.201	38.033	11.516
142	Lung cancer	1.574	56.700	29.913
143	Lung cancer	1.488	52.400	2.169
144	Lung cancer	1.254	40.717	35.746
145	Lung cancer	1.050	30.500	35.821
146	Lung cancer	1.429	49.467	39.859
147	Lung cancer	1.297	42.867	16.452
148	Lung cancer	1.340	45.000	34.250
149	Lung cancer	1.046	30.317	29.763

150	Lung cancer	1.223	39.150	21.313
151	Hepatic cancer	1.195	37.750	23.275
152	Hepatic cancer	1.667	61.350	24.476
153	Hepatic cancer	1.500	53.000	NA
154	Hepatic cancer	1.579	56.933	8.859
155	Hepatic cancer	1.427	49.350	12.388
156	Hepatic cancer	1.380	47.017	55.184
157	Hepatic cancer	1.769	66.433	19.220
158	Hepatic cancer	1.589	57.450	36.789
159	Hepatic cancer	1.600	58.000	20.647
160	Hepatic cancer	1.160	35.983	28.755
161	Hepatic cancer	1.639	59.967	13.214
162	Hepatic cancer	1.544	55.217	31.609
163	Hepatic cancer	1.806	68.317	31.909
164	Hepatic cancer	1.449	50.467	14.716
165	Hepatic cancer	1.200	38.017	13.589
166	Hepatic cancer	1.683	62.150	4.355
167	Hepatic cancer	1.858	70.917	NA
168	Hepatic cancer	1.539	54.933	47.751
169	Hepatic cancer	1.532	54.617	6.907
170	Hepatic cancer	1.264	41.200	22.148
171	Hepatic cancer	1.666	61.317	9.385
172	Hepatic cancer	1.432	49.617	5.556
173	Hepatic cancer	1.665	61.233	35.438
174	Hepatic cancer	1.238	39.900	32.660
175	Hepatic cancer	1.240	40.000	41.444
176	Hepatic cancer	2.132	84.600	27.779
177	Hepatic cancer	1.846	70.317	7.283
178	Hepatic cancer	1.765	66.250	35.663
179	Hepatic cancer	1.405	48.267	20.722
180	Hepatic cancer	1.129	34.433	36.339
181	Hepatic cancer	1.377	46.850	19.596
182	Hepatic cancer	1.282	42.083	26.879
183	Hepatic cancer	1.535	54.733	37.089
184	Hepatic cancer	1.449	50.450	0.450
185	Hepatic cancer	1.220	39.017	27.629
186	Hepatic cancer	1.322	44.100	20.572
187	Hepatic cancer	1.932	74.617	13.589
188	Hepatic cancer	1.606	58.300	57.661
189	Hepatic cancer	1.410	48.517	9.535
190	Hepatic cancer	1.640	60.000	49.402
191	Hepatic cancer	1.461	51.050	16.518
192	Hepatic cancer	1.471	51.533	41.219
193	Hepatic cancer	1.574	56.700	42.420
194	Hepatic cancer	1.076	31.817	47.600
195	Hepatic cancer	1.673	61.667	NA
196	Hepatic cancer	1.487	52.333	10.436
197	Hepatic cancer	1.478	51.883	28.380
198	Hepatic cancer	1.512	53.617	36.263
199	Hepatic cancer	1.422	49.117	24.326
200	Hepatic cancer	1.285	42.250	28.080

201	Hepatic cancer	1.371	46.567	44.898
202	Hepatic cancer	1.516	53.817	30.933
203	Hepatic cancer	1.706	63.317	31.233
204	Hepatic cancer	1.766	66.283	25.527
205	Hepatic cancer	1.567	56.367	18.019
206	Hepatic cancer	1.234	39.717	49.778
207	Hepatic cancer	1.223	39.133	18.845
208	Hepatic cancer	1.465	51.250	NA
209	Hepatic cancer	1.454	50.700	0.225
210	Hepatic cancer	1.663	61.167	3.304
211	Hepatic cancer	1.357	45.850	1.201
212	Hepatic cancer	1.678	61.883	39.041
213	Hepatic cancer	1.276	41.783	10.736
214	Hepatic cancer	1.659	60.950	24.926
215	Hepatic cancer	1.196	37.800	31.083
216	Hepatic cancer	1.766	66.317	52.856
217	Hepatic cancer	1.429	49.467	43.171
218	Hepatic cancer	1.403	48.133	22.224
219	Hepatic cancer	1.164	36.200	31.834
220	Hepatic cancer	1.563	56.150	21.698
221	Hepatic cancer	1.436	49.800	42.645
222	Hepatic cancer	1.768	66.400	26.879
223	Hepatic cancer	1.433	49.650	24.476
224	Hepatic cancer	1.230	39.500	24.401
225	Hepatic cancer	1.411	48.567	46.399
226	Hepatic cancer	1.639	59.950	22.674
227	Hepatic cancer	1.276	41.783	37.089
228	Hepatic cancer	1.643	60.150	39.942
229	Hepatic cancer	1.634	59.683	14.040
230	Hepatic cancer	1.907	73.367	14.190
231	Hepatic cancer	1.596	57.817	16.142
232	Hepatic cancer	1.836	69.783	0.375
233	Hepatic cancer	1.807	68.367	24.476
234	Hepatic cancer	1.855	70.733	7.733
235	Hepatic cancer	1.751	65.533	0.751
236	Hepatic cancer	1.746	65.317	NA
237	Hepatic cancer	1.982	77.083	14.190
238	Hepatic cancer	1.408	48.417	7.057
239	Hepatic cancer	1.384	47.183	42.795
240	Hepatic cancer	1.903	73.167	52.406
241	Hepatic cancer	1.849	70.450	0.450
242	Hepatic cancer	1.475	51.750	24.251
243	Hepatic cancer	1.741	65.067	NA
244	Hepatic cancer	1.440	49.983	32.134
245	Hepatic cancer	1.140	35.000	48.201
246	Hepatic cancer	0.995	27.767	16.442
247	Hepatic cancer	1.236	39.800	20.947
248	Hepatic cancer	1.441	50.050	18.845
249	Hepatic cancer	1.378	46.900	15.466
250	Hepatic cancer	1.134	34.683	16.142
251	Hepatic cancer	1.403	48.150	27.029

252	Hepatic cancer	1.255	40.767	37.465
253	Hepatic cancer	1.756	65.800	4.655
254	Hepatic cancer	1.752	65.600	1.126
255	Hepatic cancer	1.354	45.700	17.569
256	Hepatic cancer	1.210	38.517	34.161
257	Hepatic cancer	1.478	51.883	9.085
258	Hepatic cancer	2.123	84.150	5.105
259	Hepatic cancer	1.605	58.267	42.870
260	Hepatic cancer	1.113	33.633	26.278
261	Hepatic cancer	1.455	50.767	1.952
262	Hepatic cancer	1.331	44.567	1.727
263	Hepatic cancer	1.424	49.200	33.035
264	Hepatic cancer	1.144	35.200	12.989
265	Hepatic cancer	1.501	53.050	29.656
266	Hepatic cancer	1.284	42.200	30.482
267	Hepatic cancer	1.655	60.750	25.527
268	Hepatic cancer	1.639	59.950	27.479
269	Hepatic cancer	1.385	47.233	58.712
270	Hepatic cancer	1.337	44.850	12.538

NA* = negative reading.

Figure S5. MTC reaction colors.

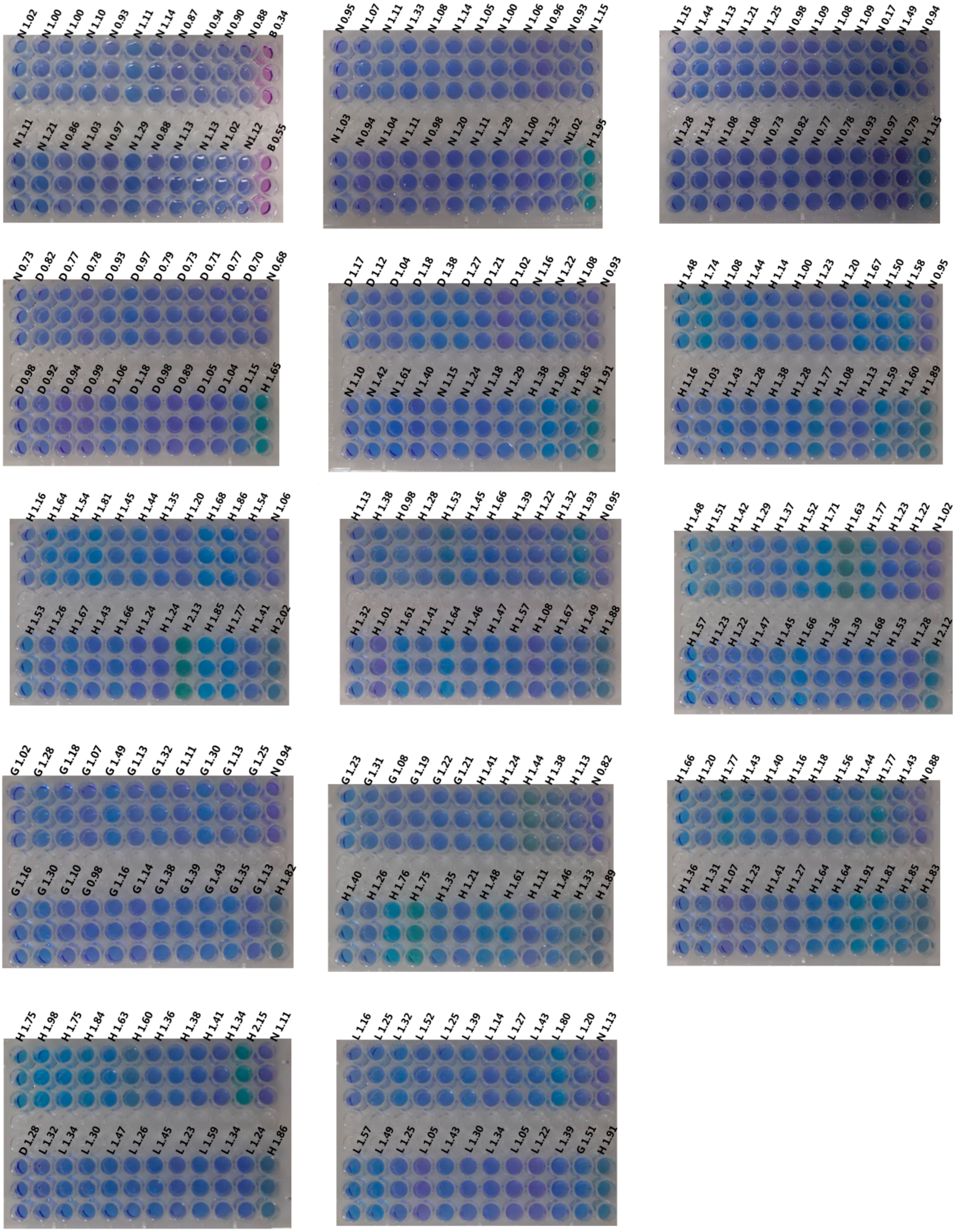

L = Lung, H = Hepatic, N = Normal, G = Gastrointestinal, D = Diabetes and B = Blank tests.
